# A cytotoxicity and comparative antibacterial study on the effect of *Zataria multiflora *Boiss, *Trachyspermum copticum* essential oils, and Enrofloxacin on *Aeromonas hydrophila*

**Published:** 2012

**Authors:** Hassan Malekinejad, Behnaz Bazargani-Gilani, Amir Tukmechi, Hadi Ebrahimi

**Affiliations:** 1 *Department of Pharmacology & Toxicology, Faculty of Veterinary Medicine, Urmia University, Urmia, I. R. Iran *; 2 *Department of Pathobiology & Quality Control, Artemia & Aquatic Animal Research Institute, Urmia University, Urmia, I. R. Iran*

**Keywords:** Natural Antibacterial Substances, Microbroth dilution Method, MTT Technique, Cytotoxicity

## Abstract

**Objective:** this study designed to test the antibacterial potency of enrofloxacin (ENR) and essential oils from *Zataria multiflora *Boiss (ZEO) and *Trachyspermum copticum* (TEO) on *Aeromonas hydrophila*.

**Material and Methods:** The antibacterial potency of test compounds was determined by several methods including the inhibition zone diameter determination, microbroth dilution method and colorimetric method of MTT. The cytotoxicity of test substances was assessed on Chinook salmon (*Oncorhynchus tshawytscha*) embryo **(CHSE**-214) **cells**.

**Results:** Results showed that ENR and tested essential oils exert antibacterial effect against *A. hydrophila*. Moreover, ENR exerted the most potent antibacterial effect with MIC values of 62.5 ng/ml. The natural compounds of ZEO and TEO also showed antibacterial effects with rather high MIC values of 0.315 mg/ml, and 1.25 mg/ml, respectively. None of the tested substances showed toxicity on CHSE-24 cells.

**Conclusion:** It is concluded that ZEO and TEO could be applied to prevent from *A. hydrophila* infection. Moreover, data also suggest that MTT method could be both cost- and time-effective and accurate method of MIC determination.

## Introduction

*Aeromonas* species are Gram-negative and anaerobic bacteria which are found in sea, river, fresh, and ground water. There are accumulating data indicating that *Aeromonas hydrophila (A. hydrophila)* is the causative agent for several diseases in cold-blooded animals including fish and reptiles and in warm-blooded animals such as mammals and birds (Austin et al., 1996 ▶; Kuhn et al., 1997 ▶). In humans, *A. hydrophila *causes gastroenteritis and bacteremia and in fish produces hemorrhagic septicemia (Chen et al., 2010 ▶; Clinical and Laboratory standards Institute [CLSI], 2008 ▶). Moreover, there are reports indicating that *A. hydrophila* in particular is one of the foodborne pathogens in seafood and shellfish and in foods from other sources such as raw red meat, poultry, dairy products, and vegetables (Ko et al., 2000 ▶; Soler et al., 2002 ▶; Daskalov, 2006 ▶).

*Zataria multiflora *Boiss (*Z. multiflora*), belongs to the family of *Labiatae* and it is a native plant to Iran, Pakistan, and Afghanistan. *Z. multiflora* with the vernacular name of Avishan-e-Shirazi in Iran is used traditionally in foods such as yogurt and pizzas for its strong and pleasant aroma. It is traditionally used as antiseptic, anesthetic, and antispasmodic. *Z. multiflora has also *shown to have analgesic and anti-inflammatory effects (Hosseinzadeh et al., 2000 ▶; Ramezani et al., 2005 ▶). The immunostimulatory effects of *Z. multiflora *essential oils in common carps has been recently reported (Soltani et al., 2010 ▶).

*Trachyspermum copticum* (*T. copticum*) as a household medicinal plant is traditionally used against gastrointestinal disorders such as indigestion and colic pain (Kaur and Arora, 2009 ▶). It is also used as a spice and preservative in food. Essential oil of the *T. copticum* (ajowan) exerts carminative and diuretic effect as well. Ajowan although is native to India, it is also cultivated in Iran, Egypt, Pakistan, and Afghanistan. In this regard, it has been shown that the essential oil of *T. copticum* could be safely used to protect the foods from toxicogenic fungi invasion (Rasuli et al., 2008 ▶). 

The susceptibility of *A. hydrophila* to various antibacterial agents including ciprofloxacin has been previously reported (Felmingham and Robbins, 1992 ▶). However, little is known about the antibacterial effect of enrofloxacin (ENR) on this bacterium. At the same time, to the best of our knowledge, there is lack of data about the antibacterial effect of *Z. multiflora* and *T. copticum* essential oils against *A. hydrophila*. Hence, this study was designed to show the potential antibacterial effect of two important and traditionally used medicinal plant essential oils in parallel to ENR as a known member of quinolones. As the selected bacterium for investigation is found predominantly in aquatic environments, to show the safety of test compounds on target animals, we studied the cytotoxicity of both essential oils on CHSE-214 cells which were originally isolated from the Chinook salmon (*Oncorhynchus tshawytscha*) embryo. Moreover, the efficacy of recently developed method of MTT in MIC determination was also evaluated.

## Material and Methods

**Extraction of essential oils**

The plant, *Zataria multiflora *Boiss (Labiatae) was purchased from local grocery and authenticated at Institute of Medicinal Plants, Karaj, Iran. A voucher specimen was preserved in the herbarium of the same institute (herbarium no: 21-13). *Trachyspermum copticum *was collected from east of Iran and was kindly identified by an expert botanist in the herbarium of Urmia university, Urmia, Iran (herbarium no: 293-0303-1). The dried aerial parts of *Z. multiflora *and ground seeds of *T. copticum* were subjected to hydrodistillation for 2.5-3 h using a Clevenger-type apparatus. The extracted oil was dried over anhydrous sodium sulfate, filtered and stored at 4 °C before use. Yield of essential oil (v/w) was calculated as volume of collected oil/weight of dry starting material ×100. The extraction yield was 3.7% and 1.9% for *Z. multiflora *and *T. copticum, *respectively. 

**Bacterial strain and drug preparation**

The bacterial strain of *Aeromonas hydrophila* (BCCG/LMG 3740) was used in the current study. Bacterium was cultured for 18 h at 28 °C in brain heart infusion broth (Merck, Darmstadt, Germany) aerobically, and used as inoculum. Enrofloxacin (VETRANAL^®^, analytical standard, Fluka). *Z. multiflora *(ZEO) and *T. copticum* essential oils (TEO) were dissolved in dimethyl sulfoxide (DMSO) as stock solution and the working solution of the compounds were freshly prepared as the ultimate concentration of the solvent which did not exceed from five percent of the final volume of the culture medium. 

**Determination of the Inhibition zone diameter**

In order to screen the antibacterial effect of ENR, ZEO and TEO and to measure the diameter of inhibition zone, we used the punch hole method. In short, the surface of previously prepared Muller-Hinton agar medium (Merck, Darmstadt, Germany) was inoculated with a 10^7^ *CFU*/ml. After 10 min, the surface of the agar medium was punched using a sterile cork borer. Twenty µl from individual test compounds at various concentrations were dropped into the holes and kept in an incubator for 24 h at 28 ^◦^C. The diameter of each clear inhibition zone was measured with digital caliper in triplicate and expressed in mm. 

**MIC determination by standard microbroth dilution method**

To determine the MIC values of ENR, ZEO, and TEO on selected strain of bacterium, the latest standard method of microbroth dilution, which has been described by the Clinical and Laboratory Standards Institute (CLSI), with minor modifications, was applied. A density of 5×10^4^ *CFU*/well was prepared in 96-well micro-titer plate containing various concentrations of ENR (3.9, 7.8, 15.6, 31.25, 62.5, 125, 250, and 500 ng/ml), ZEO (0.07, 0.15, 0.31, 0.62, 1.25, 2.5, 5, and 10) or TEO (0.07, 0.15, 0.31, 0.62, 1.25, 2.5, 5, and 10 mg/ml) in 100 µl/well of the Mueller-Hinton broth (Merck, Darmstadt, Germany) medium. 

The culture medium alone and medium containing bacteria without test compounds were considered as two controls. Triplicate wells were applied for each concentration of the individual test materials. The inoculated wells were incubated at 28 °C for 9 h. the turbidity of the medium was measured by a microplate reader (Stat Fax 2100, USA) at 630 nm. The MIC value was determined as the concentration, which the measured absorbance was equal or lower than that of the controls (wells that received no bacteria). 

**MIC determination by MTT method**

To compare the MTT method with routine method of microbroth dilution, the same density of the *A. hydrophila* was seeded in the same culture medium and the concentrations of ENR, ZEO, and TEO, were provided for MTT method. The culture medium alone and medium containing bacteria without test compounds were used as controls. The plates were incubated for 9 h at 28 °C in aerobic conditions. A concentration of 5 mg/ml MTT [3-(4, 5-dimethylthiazol-2-yl)-2, 5-diphenyltetrazolium bromide] was prepared in PBS (pH 7.2). Twenty µl of MTT solution was added to each single well and the 96-well micro-titer plates were incubated for 30 min at 28 ºC. After 30 min incubation with MTT solution, to avoid losing the formed formazan granules, 80% of the MTT solution was carefully removed, the insoluble purple formazan granules were solubilized with MTT lyses buffer (0.5% sodium dodecyl sulphate, 36 mM HCl, and isopropanol acid) and the absorbance was measured at 540 nm. In order to optimize the incubation time of the bacterial suspension with MTT solution, several incubation time periods (5, 10, 15, 20, 25, and 30 min) were tested. The ultimate absorbance for each well was calculated as: 

Ab_ (540 nm) _of the sample–Ab_ (540 nm)_ of the control;

Where the control contains only the culture medium and test compound solvent. 

**Cell culture and effect of essential oils and ENR on CEHS-214 cells**

The Chinook salmon (*Oncorhynchus tshawytscha*) embryo (CHSE-214) cells were grown in DMEM supplemented with 10% FCS, 1% penicillin (100 units/ml), streptomycin (100 µg/ml), and 1% L-glutamine. Cells were sub-cultured every 3-4 days and remained at 22 °C in a humidified atmosphere of 5% CO_2_ in air. For cell viability assay, cells were seeded in 96-well tissue culture plates at a density of 2×10^4^ cells/well and in 0.2 ml medium. Cells were incubated for 24 hours prior to exposure to test chemicals.

**Cell viability test**

Cell viability was quantified by the colorimetric MTT assay. This assay measures the reduction of dimethylthiazol diphenyl tetrazolium bromide (MTT; stock solution 3 mg/ml in PBS) to formazan by the mitochondrial enzyme succinate dehydrogenase. This reduction capacity reflects the number of viable cells. Following 24 h exposure of the cells against various concentrations of test compounds, the medium was discarded and 0.2 ml MTT solution was added to the cells. After 3 h incubation at 37°C, the MTT solution was discarded and the intracellular purple insoluble formazan was solubilized by adding 100 µL/well of lysis buffer. Following shaking on orbital shaker, the optical density (OD) was measured at 540 nm using a micro plate reader (Stat Fax 2100, USA).

**Statistical analyses**

Results are expressed as mean±S.D. The data was analyzed by one-way ANOVA followed by bonferroni post hoc test for multiple comparisons using Graph Pad Prism software (version 2.01. Graph Pad software Inc. San Diego, California). A p-value less than 0.05 was considered significant. 

## Results

**Determination of the diameter of inhibition zone**

Our primary results from agar well diffusion assay indicated that ENR, ZEO, and TEO were able to exert an antibacterial effect on the selected strain of *A. hydrophila*. ENR and TEO were found to be the strongest and weakest antibacterial agents, respectively. Moreover, this assay also revealed that the antibacterial effect of test compounds is concentration-dependent. The diameter of inhibition zone for ENR, ZEO, and TEO are presented in Table 1. 

**Table 1:** Antibacterial characters of ENR, ZEO and TEO on *A. hydrophila*.

**Test compounds**	**Inhibition Zone (mm)**	**MIC**
ENR (5 ng/ml)	9.0±0.7	62.5 ng/ml
ZEO (0.1 mg/ml)	35.0±1.9	0.315 mg/ml
TEO (0.1 mg/ml)	27.0±3.1	1.25 mg/ml

**MIC determination by** **microdilution** **and MTT method**

**Figure 1:**
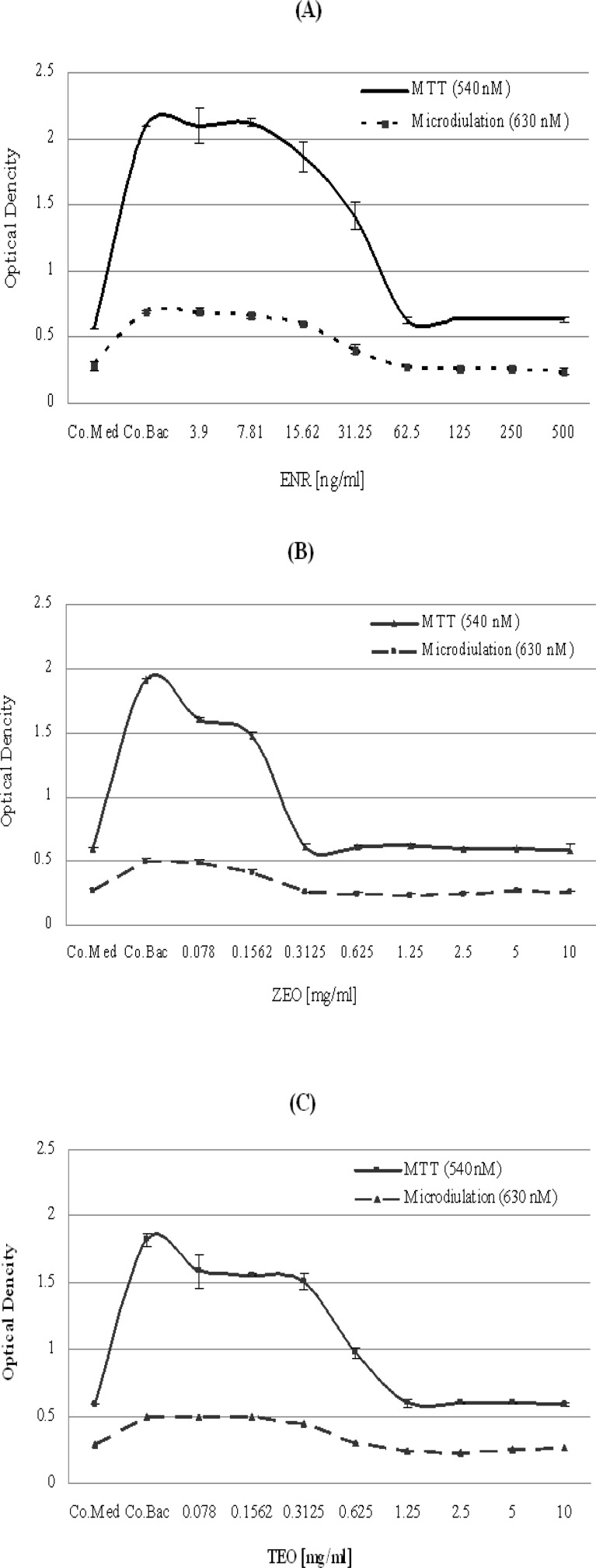
Antibacterial effect of (**A**): Enrofloxacin, (**B**): *Z. multiflora *(ZEO) and (**C**) *T. copticum* essential oils (TEO) against *A. hydrophila*. Antibacterial effect was tested by using two methods of microbroth dilution and MTT. Co Med =Control medium and Co Bac=Control Bacteria

In order to determine the MIC values of the test compounds we conducted two methods: the standard microdilution method which is based on the assessment of produced turbidity upon bacterial density and MTT method, which has been recently developed as a colorimetric assay. Figure 1 (A, B and C) shows the antibacterial potency of test compounds using both methods. For all three compounds, the MTT method showed a high sensitivity compared with microdilution assay. ENR exerted an excellent antibacterial effect and ZEO and TEO showed antibacterial property against *A. hydrophila*. The estimated MIC values are depicted in Table 1. 

**Essential oils and ENR did not show**

**cytotoxic effect on CHSE cells**

In order to examin the cytotoxic effect of tested compounds, the Chinook salmon (*Oncorhynchus tshawytscha*) embryo (**CHSE**-214) **cells** were exposed for 24 h to test substances at similar concentrations to the antibacterial assay. Cell viability was assessed by MTT test and the results did not show any toxicity on cells. 

## Discussion

The results of antibacterial determination performed by all three techniques of inhibition zone assay, microbroth dilution, and MTT, showed that the tested essential oils exerted remarkable antibacterial activity against *A. hydrophila*. Therefore, they could be introduced as natural antibacterial agents against commonly occurring microorganism in aquatic environments including drinking water. Previous studies reported the antibacterial effect of *Z. multiflora *Boiss essential oil against various bacteria such as *Salmonella typhimurium*,* Staphylococcus aurous* and* Bacillus cereus* in a food model system and in brain heart infusion (BHI) broth (Fazeli et al., 2007 ▶; Misaghi and Akhondzadeh, 2007 ▶; Moosavy et al., 2008 ▶). As reported by many researchers, ZEO antimicrobial activity against pathogenic microorganisms attribute to the high content of oxygenated monoterpens, particularly thymol, carvacrol, P-cymene, and relatively high percentage of γ-Terpinene (Ebrahimzadeh et al., 2003 ▶).

Our MIC determination assays indicated that TEO also exerted antibacterial effect against *A. hydrophila*. Although the MIC value for TEO in comparison to ZEO was found four-fold higher, but, its antibacterial effect on *A. hydrophila *is comparable with currently used chemical agents such as ampicillin, chloramphenicol, and gentamicin. Antibacterial property of aqueous and organic extracts of *T. copticum* against Gram-positive bacteria such as *S. aurous* and Gram-negative bacteria including *E. coli* has been reported (Kaur et al., 2009 ▶). Previous analytical studies reported that the essential oil from *T. copticum* mainly contains thymol (37.2%), p-cymene (32.3%), γ-terpinene (27.3%), β-pinene (1%) and α-pinene (0.6%), which the antibacterial property of TEO likely attributes to the mentioned substances (Rasooli et al., 2008 ▶). As traditionally essential oil from *T. copticum* is used for abdominal pain relief particularly for carminative purposes, it may be explained by these findings that the active compounds of ajowan lead to the reduction of gas producing bacteria and ultimately relieving the bacteria-induced pain. 

Enrofloxacin belongs to the super-family of fluoroquinolone antibiotics, which were originally developed against the Gram-negative aerobic bacteria. There are, however, studies showing that the newer generation compounds are also effective against some Gram-positive pathogens (Blondeau et al., 2012 ▶; Farca et al., 2007 ▶). In this study, we showed that ENR exerted an excellent antibacterial effect on *A. hydrophila* as a Gram-negative and anaerobic bacterium with MIC value of 62.5 ng/ml. Therefore, it would worth to investigate the pharmacokinetic characteristics of ENR to support its implication in aquatic environment. In this regard, previous studies showed that the addition of ENR to aquatic systems had a minimal effect on water quality and microbial communities (Knapp et al., 2005 ▶).The MIC value of 62.5 ng/ml for ENR which was obtained in this study, in comparison with the previous reports (250 mg/l), is rather low and it might be related either to the high sensitivity of used strain of the bacterium and/or to the high sensitivity of used method for MIC determination. Previous reports showed that ciprofloxacin as another member of fluoroquinolone family exert an excellent antibacterial activity against *A. hydrophila* in drinking water (Scoaris et al., 2008 ▶). In order to examine the cytotoxic effect of tested compounds, the Chinook salmon (*Oncorhynchus tshawytscha*) embryo (**CHSE**-214) **cells** were exposed for 24 h to individual substance at similar concentrations for antibacterial assay. Cell viability was assessed by MTT test and significant cytotoxicity has not been observed. 

The test compounds including chemical agent and natural essential oils not only showed the antibacterial effect against *A. hydrophila *but also showed no cytotoxicity against **CHSE**-214 Chinook salmon embryo** cells**. Therefore, it would be interested to take in consideration the further pharmacokinetic parameters of these compounds in order to prove the application of natural substances in aquaculture and aquatic originated foods.

Another finding of the current study was illustrating the high efficiency of MTT technique in determining MIC as a newly developed substance. We showed that using the MTT method, MIC values of test compounds are determined more accurately and rapidly. Recently, the MTT assay as a colorimetric method has been introduced to test the antibacterial susceptibility of various synthetic and natural remedies. This method is a cost-effective and rapid technique (Raut et al., 2008 ▶; Shi et al., 2008 ▶).

This study reports remarkable antibacterial effects of ENR as chemical agent and ZEO and TEO as natural substances against the most commonly existing strain of *Aeromonas* in aquaculture. As none of the tested compounds showed cytotoxicity against **CHSE**-214 cells, thus the compounds could be valuable candidates for further evaluation of this substance for prevention and protection of aquatic environments and foods from *A. hydrophila *contamination. Additionally, the high efficiency of MTT technique in comparison with routinely used microbroth dilution and disk diffusion methods in MIC determination was also demonstrated. Therefore, it would be a more accurate and time- and cost-saving technique to test the antimicrobial potency of various new natural compounds.
